# Sign Language Recognition with the Kinect Sensor Based on Conditional Random Fields

**DOI:** 10.3390/s150100135

**Published:** 2014-12-24

**Authors:** Hee-Deok Yang

**Affiliations:** Department of Computer Engineering, Chosun University, Seosuk-dong, Dong-ku, Gwangju 501-759, Korea; E-Mail: heedeok_yang@chosun.ac.kr; Tel.: +82-62-230-7474.

**Keywords:** sign language recognition, conditional random field, BoostMap embedding

## Abstract

Sign language is a visual language used by deaf people. One difficulty of sign language recognition is that sign instances of vary in both motion and shape in three-dimensional (3D) space. In this research, we use 3D depth information from hand motions, generated from Microsoft's Kinect sensor and apply a hierarchical conditional random field (CRF) that recognizes hand signs from the hand motions. The proposed method uses a hierarchical CRF to detect candidate segments of signs using hand motions, and then a BoostMap embedding method to verify the hand shapes of the segmented signs. Experiments demonstrated that the proposed method could recognize signs from signed sentence data at a rate of 90.4%.

## 1. Introduction

Sign language is a visual language used by deaf people, which consists of two types of action: signs and finger spellings. Signs are dynamic gestures characterized by continuous hand motions and hand configurations, while finger spellings are static postures discriminated by a combination of continuous hand configurations [1–3]. The term “gesture” means that the character is performed with hand motions, while “posture” refers to a character that can be described with a static hand configuration [4]. Sign language recognition has been researched using various input devices, such as color cameras, stereo cameras, data gloves, Microsoft's Kinect sensor, time of flight (TOF) cameras, *etc.* [5]. Although the data glove-based sign language recognition systems have achieved better performance than other systems, data gloves are too expensive and too uncomfortable to use, which limits their popularity.

Several approaches to sign language recognition acquire information from range sensors such as TOF cameras or the Kinect, which was developed to interact with video games as a means for full-body tracking of body movements and gestures [6]. Many researchers have developed applications with gesture and sign language recognition systems using these sensors such as interactive displays [7], physical rehabilitation [8], robot guidance [9,10], gesture recognition [11], sign language recognition [12,13], hand gesture recognition [14], *etc.*

Depth information-based sign language recognition has become more widespread because of improved interactivity, and user comfort, and the development of consumer-priced depth sensors, such as Microsoft's Kinect [5]. Depth information-based approaches are generally more accurate and can recognize a wider vocabulary than color or 2D-based approaches.

Numerous studies have attempted to use the Microsoft Kinect to identify hand gestures. Zafrulla *et al.* investigated the potential of the Kinect depth-mapping camera for sign language recognition [12]. They collected a total of 1000 American Sign Language (ASL) phrases and used a hidden Markov model (HMM) to recognize the signed phrases. Ren *et al.* researched a robust hand gesture recognition system using a Kinect [5]. They proposed a modified Finger-Earth Mover's Distance metric (FEMD) in order to distinguish noisy hand shapes obtained from the Kinect sensor. They achieved a 93.2% mean accuracy on a 10-gesture dataset.

Chai *et al.* proposed a sign language recognition and translation system based on 3D trajectory matching algorithms in order to connect the hearing impaired community with non-hearing impaired people [13]. They extracted 3D trajectories of hand motions using the Kinect, and collected a total of 239 Chinese sign language words to validate the performance of the proposed system. They achieved rank-1 and rank-5 recognition rates of 83.51% and 96.32%, respectively. Moreira Almeida *et al.* also proposed a sign language recognition system using a RGB-D sensor. They extracted seven vision-based features from RGB-D data, and achieved an average recognition rate of 80% [15].

In addition to the Kinect, other methods of recognizing hand gestures have also been explored. Shotton predicted 3D positions of body joints from a single depth image without using temporal information [16]. Palacois *et al.* proposed a system for hand gesture recognition that combined RGB and 3D information provided by a vision and depth sensor, the Microsoft Asus Xtion Pro Live [6]. This method, using a defined 10-gesture lexicon, used maximums of curvature and convexity defects to detect fingertips.

Additional methods for hand movement recognition include a study by Lahamy and Lichti that used a range camera to recognize the ASL alphabet [4]. A heuristic and voxel-based signature was designed and a Kalman filter was used to track the hand motions. This method proposed a rotation invariant 3D hand posture signature. They achieved a 93.88% recognition rate after testing 14,732 samples of 12 postures taken from the ASL alphabet. In addition, Yang *et al.* [1–3] used a threshold model with a CRF, which performed an adaptive threshold for distinguishing between in-vocabulary signs and out-of-vocabulary non-signs. They proposed augmenting the CRF model by adding one additional label to overcome the weaknesses of the fixed threshold method.

In this paper, we focus on recognizing signs in a signed sentence using 3D information. The difficulty of sign language recognition comes from the fact that sign occurrences vary in terms of hand motion, shape, and location. The following three problems are considered in this research: (1) signs and non-sign patterns are interspersed within a continuous hand-motion stream; (2) some signs shares patterns; and (3) each sign begins and ends with a specific hand shape.

In order to solve the first and second problems, a hierarchical CRF (H-CRF) is applied [2]. The H-CRF can discriminate between signs and non-sign patterns using both hand motions and hand locations. The locations of the face and both hands are needed to extract features for sign language recognition. The subject's 3D upper-body skeletal structure can be inferred in real-time using the Kinect. Information about body components in 3D allows us to locate various structural feature points on the face and hands. The H-CRF can recognize the shared patterns among the signs. An error in the middle of a sign implies that the sign has been confused with another sign because of the shared patterns, or an improper temporal boundary has been detected.

In order to solve the third problem, BoostMap embeddings are used to recognize the hand shapes. The BoostMap embeddings are robust to various scales, rotations, and sizes of the signer's hand, which makes this method ideal for this application. The main goal of this hand shape verification method is to determine whether or not to accept a sign spotted by means of the H-CRF. This helps to disambiguate signs that may have similar overall hand motions but different hand shapes.

**Figure 1.**
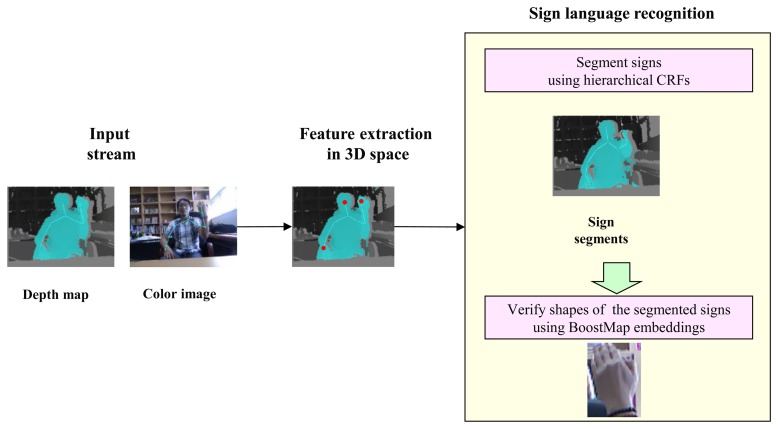
Overview of the proposed method for recognizing a sign.

Figure 1 shows the framework of our sign language recognition system. We use the Kinect, which acquires both a color image and its corresponding depth map. The hand and face locations are robustly detected in varying lighting conditions. After detecting the locations of the face and hands, an H-CRF is used to detect candidate sign segments using hand motions and locations. Then, the BoostMap embedding method is used to verify the hand shapes of the segmented signs.

## 2. Sign Language Recognition

### 2.1. Face and Hand Detection

**Figure 2.**
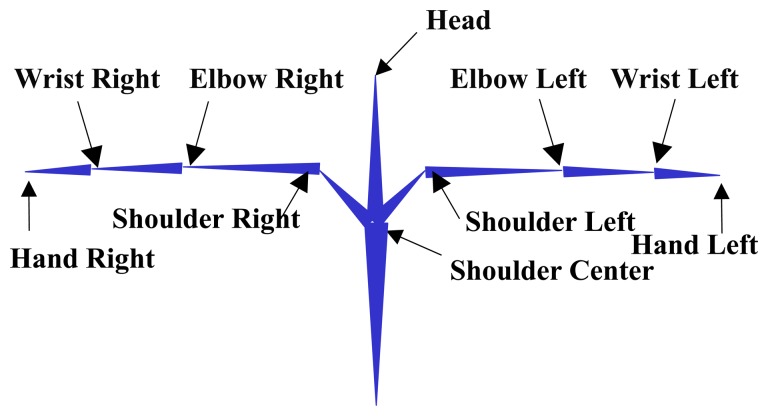
Skeleton model: 10 upper body components.

The face and hand positions are robustly detected using the hand tracking function in the Kinect Windows software development kit. The skeletal model consists of 10 feature points that are approximated from the upper body as shown in Figure 2.

**Figure 3.**
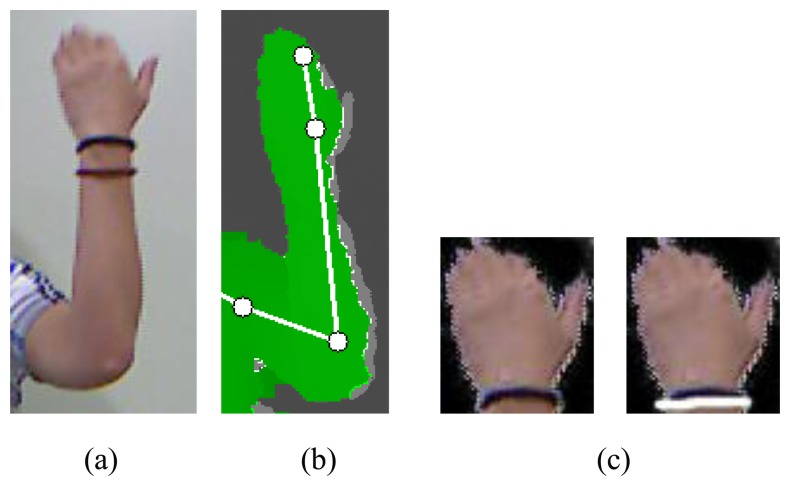
Hand detection: (**a**) color image (**b**) depth image and feature positions (**c**) and detected regions with black wristband.

The hand region is obtained by establishing a threshold from the hand position as shown in Figure 3a. The signer wears a black wristband to segment the hand shape [5]. RANdom SAmple Consensus (RANSAC) [17] is used to detect the black wristband, as shown in Figure 3c. The detected hand shape is normalized.

### 2.2. Feature Extraction

Six and one features are extracted in 3D and 2D space, respectively, using the detected hand and face regions as shown in Table 1 [1–3].

The feature, *HF_L_*, represents the location of the left hand with respect to the signer's face in 3D space. The distance between the face and left hand, *D_HFL_*, and the angle from the face to the left hand, *θ_HFL_*, is measured. In order to extract 3D features, the coordinates of the left hand are projected into the *x*, *y* and *z* axes. The angle between the face and left hand, *θ_HFL_* = {*θ_x_*, *θ_y_*, *θ_z_*}, is extracted. Then, the feature vector {*D_HFL_*, *θ_HFL_*} is clustered into an index using an expectation-maximization (EM)-based Gaussian Mixture Model (GMM) [1]. Features, *HH_L_*, *HF_R_*, *HH_R_*, *FS_L_* and *FS_R_*, are likewise calculated and clustered.

The hand occlusion, *OH_LR_*, is determined from the ratio of the overlapping regions of the two hands in the frontal view:

(1) $$OH_{LR}=\{\begin{array}{cc} 1, & \min\left(\frac{R_{o}}{H_{l}},\frac{R_{o}}{H_{r}}\right)>T_{o},\\ 0, & \text{otherwise}, \end{array}$$

where *H_l_* is the left hand region, *H_r_* is the right hand region, *R_o_* is the overlapping region between the two hands, and *T_o_* is the threshold for hand occlusion (*T_o_* = 0.3, as determined by experimentation).

### 2.3. CRF-Based Sign Language Recognition

A hierarchical CRF framework is used to recognize the sign language [2]. In the first step, a threshold model (T-CRF) is used to distinguish between signs and non-sign patterns [1]. In this step, non-sign patterns are defined by the label “N-S” and signs are defined by the labels in the vocabulary. When constructing the T-CRF, a conventional CRF is constructed first. The conventional CRF includes the labels *S_C_* = {*Y*_1_, ⋯, *Y_l_*}, where *Y*_1_ through *Y_l_* are labels for signs, and *l* is the number of signs in the vocabulary [1].

In a CRF, the probability of a label sequence y, given an observation sequence x, is found using a normalized product of potential functions. Each product of potential functions is represented by [1]:

(2) $$p_{\theta }\left(\text{y}|\text{x}\right)=\frac{1}{Z_{\theta }\left(\text{x}\right)}\exp\left(\sum_{i-1}F_{\theta }\left(y_{i-1},y_{i},\text{x},i\right)\right)$$

where *F_θ_*(*y_i_*_−1_, *y_i_*, x, *i*) = Σ*_v_ λ_v_ t_v_*(*y_i_*_−1_, *y_i_*, x, *i*) + Σ*_m_ μ_m_ s_m_*(*y_i_*, x, *i*), *t_v_*(*y_i_*−1, *y_i_*, x, *i*) is a transition feature function of the observation sequence x at positions *i* and *i* − 1, where *s_m_*(*y_i_*, x, *i*) is a state feature function of observation sequence x at position *i*, *y*_*i* − 1_ and *y_i_* are the labels of observation sequence x at positions *i* and *i* − 1, and *λ_v_* and *μ_m_* are the weights of both the transition and state feature functions, respectively. *θ* represents the weights of the transition features and state feature functions, and *Z_θ_*(x) is the normalization factor.

The feature vector x*_t_*, of the observation sequence x, at time *t*, is expressed as:

(3) $${\text{x}}_{t}=\left\{HL_{L}^{t},HR_{R}^{t},HH_{L}^{t},HH_{R}^{t},FS_{R}^{t},FS_{R}^{t},OH_{LR}^{t}\right\}$$

CRF parameter learning is based on the principle of maximum entropy. Maximum likelihood training selects parameters that maximize the log-likelihood of the training data [1]. The T-CRF is built using weights from the constructed conventional CRF. In addition, the label “N-S” for non-sign patterns is added to the conventional CRF. Thus, the T-CRF includes the labels *S_T_* = {*Y*_1_, ⋯, *Y_l_*, *N*-*S*}. The starting and ending points of in-vocabulary signs were calculated by back-tracking the Viterbi path, subsequent to a forward pass [1].

The weights of the transition feature functions from other labels to the non-sign pattern label “N-S” and vice versa are assigned by:

(4) $$\begin{array}{c} \forall_{k\in \left\{1,\ldots ,l\right\}}\lambda_{\nu }\left(Y_{k},N‐S\right)=\frac{\lambda_{\nu }\left(Y_{k},Y_{k}\right)}{l},\\ \forall_{k\in \left\{1,\ldots ,l\right\}}\lambda_{\nu }\left(N‐S,Y_{k}\right)=\frac{\lambda_{\nu }\left(N‐S,N‐S\right)}{l}, \end{array}$$

where $\lambda_{\nu }\left(N‐S,N‐S\right)=\underset{k=1,\ldots ,l}{\mathrm{argmax}}\lambda_{\nu }\left(Y_{k},Y_{k}\right)+\kappa$, and *κ* is the weight of the self-transition feature function of the non-sign pattern label “N-S” [1].

After constructing the T-CRF, *i.e.*, the first layer of the hierarchical CRF, the second layer CRF, which models common sign actions, is constructed. The output of the first layer is the input of the second layer. It contains the segmented signs, which signs have a higher probability than the non-sign pattern label “N-S”. As a result, the second layer CRF only has labels S_C_ = {*Y*_1_, ⋯, *Y_l_*}. The detailed algorithm is described in [1].

Finally, the probability of the recognized sign is calculated as:

(5) $$P\left({y_{i}}^{t}\right)=\frac{\sum_{i=S_{S}}^{S_{e}}p_{\theta }\left({y_{i}}^{t}|\text{x}\right)}{S_{e}-S_{s}+1}$$

where *p_θ_* (*y_i_^t^*|x) is the marginal probability of the sign *y_i_* at time *t*; *S_s_* and *S_e_* are the start and end frames of the segmented sign, respectively.

### 2.4. Shape-Based Sign Language Verification

The hierarchical CRF is useful for recognizing hand motions; however, it has difficulty distinguishing between different hand shapes. The main goal of the hand shape-based sign verification is to determine whether or not a sign spotted through the H-CRF should be accepted as a sign. The shape-based sign verification module is performed at the end frame of a recognized sign, when P (*y_i_^t^*) in Equation (5) is lower than a threshold.

**Figure 4.**
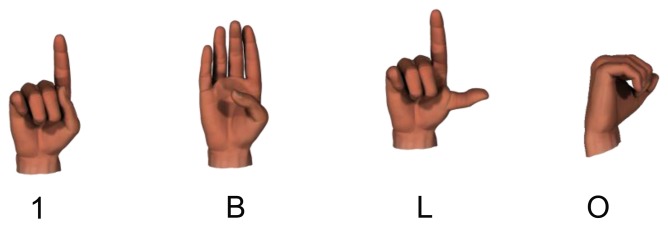
Examples hand shape used for training the BoostMap embeddings.

BoostMap embeddings are applied in order to recognize the hand shape. This method accommodates various scales, rotations, and sizes of the signer's hands [2,18]. Synthetic hand images to train the model are generated using the Poser 7 animation software. Each sign begins and ends with a specific hand shape, and each alphabet has unique hand shapes. Table 2 and Figure 4 show examples of hand shapes for sign language recognition. Our system uses a database with 17 hand shapes. For each hand shape, 864 images are generated.

The hand shapes are verified over several frames, and a detected sign is accepted when the voting value *V_s_* (*y_i_^t^*) exceeds threshold *T_s_*. The voting value, *V_s_* (*y_i_^t^*) is calculated as:

(6) $$V_{S}\left({y_{i}}^{t}\right)=\sum_{j=t-t_{a}}^{t+t_{a}}C_{a}\left({y_{i}}^{t},B\left(j\right)\right),$$

where *y_i_^t^* is the sign detected by the H-CRF at position *t*, and *t_a_* is the window size. *C_a_* (*y_i_^t^*, *B* (*j*)) is:

(7) $$C_{a}\left({y_{i}}^{t},B\left(j\right)\right)=\begin{array}{cc} 1, & {y_{i}}^{t}=B\left(j\right),\\ 0, & \text{otherwise}, \end{array}$$

where *B* (*j*) is the recognition result of the BoostMap embedding method at time *j*.

## 3. Experimental Results and Analysis

### 3.1. Experimental Environment

**Figure 5.**
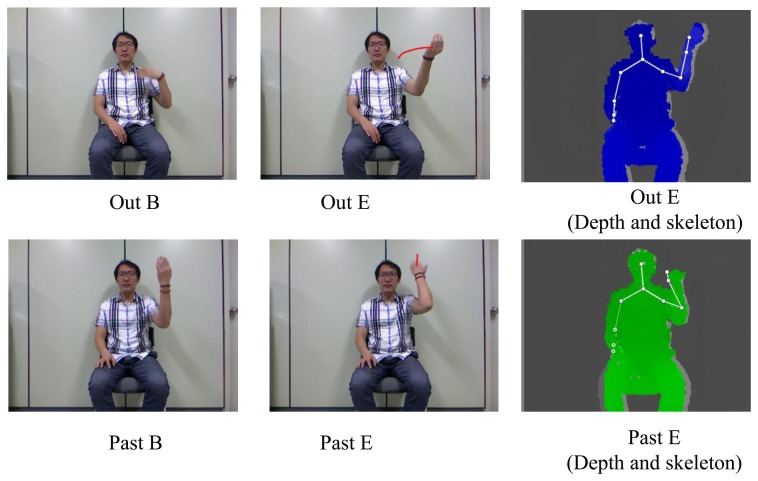
Two examples of ASL signs; B and E indicate means beginning and end, respectively.

For training the CRFs and H-CRFs, 10 sequences for each sign in the 24-sign lexicon were collected. The signer wore a black wristband during data collection. The start and end points of the ASL signs were added manually to the training data and for the ground truth, they were used for testing the performance of the proposed method. We captured the video with a Kinect device. Of the 24 signs, seven were one-handed signs, and 17 were two-handed signs, as shown in Table 3. Figure 5 shows two examples of signs used in the experiments. In general, most sign language recognition tasks face three types of errors—substitution errors, insertion errors, and deletion errors.

An insertion error occurs when the spotter reports a nonexistent sign. A deletion error occurs when the spotter fails to spot a sign in an input sequence. A substitution error occurs when an input sign is incorrectly classified [1–3]. The sign error rate (SER) and correct recognition rate (R) are calculated by:

(8) $$\begin{array}{c} \text{SER}\left(\%\right)=\frac{I+S+D}{N},\\ \text{R}\left(\%\right)=\frac{C}{N}, \end{array}$$

where N, S, I, D, and C are the numbers of signs, substitutions, insertions, and deletion errors, and correctly detected signs, respectively. An H-CRF was implemented and the results of the sign language recognition were compared to the performance accuracy in both 2D and 3D feature space.

### 3.2. Sign Language Recognition with Continuous Data

As shown in Table 4, 3D features decrease insertion and substitution errors, while slightly decreasing deletion errors, compared to the model with 2D features. As a result, the SER of the H-CRF^3D^ decreases; however, the correct recognition rates of the H-CRF^3D^ increases.

**Figure 6.**
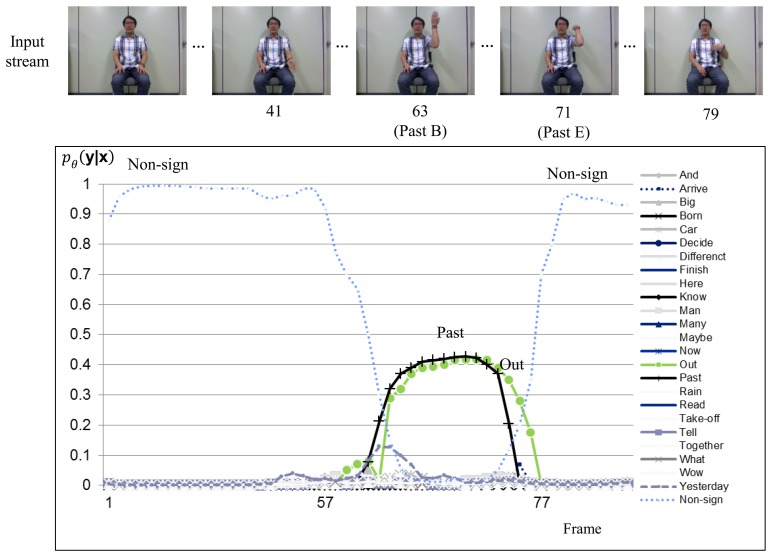
Sign language recognition result for H-CRF**^2^*^D^*** using a signed sentence.

**Figure 7.**
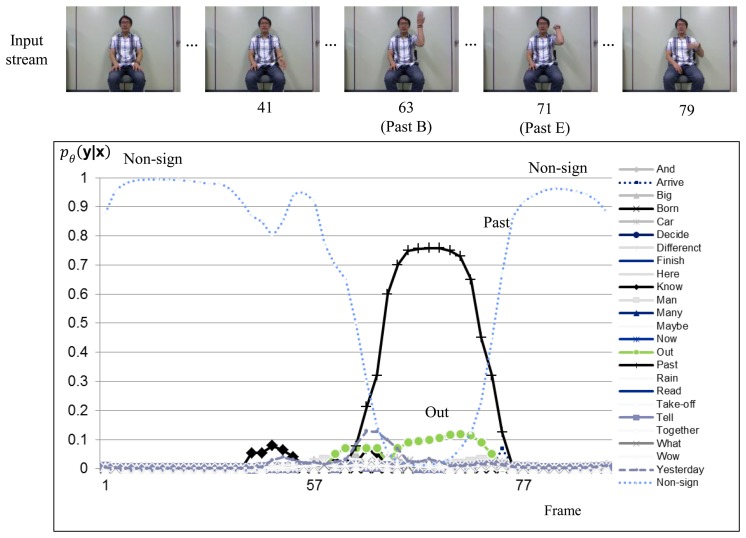
Sign language recognition result for H-CRF**^3^***^D^* using a signed sentence.

Figures 6 and 7 show sign recognition results for a sign sequence that contains two in-vocabulary signs “OUT” and “PAST” with H-CRF^2D^ and H-CRF^3D^, respectively. The time evolutions of the probabilities for in-vocabulary signs and non-sign patterns are illustrated by curves. The probabilities of the signs “OUT” and “PAST” fluctuate, while the sign is performed, as shown in Figure 6, because of the similar hand motions of these two signs in 2D space. On the other hand, as shown in Figure 7, the label for non-sign patterns has the greatest probability during the first 63 frames. Then, it is followed by the sign “OUT.” After 63 frames, the probability of the sign “OUT” nearly becomes 0.1, and there is a non-sign pattern.

**Figure 8.**
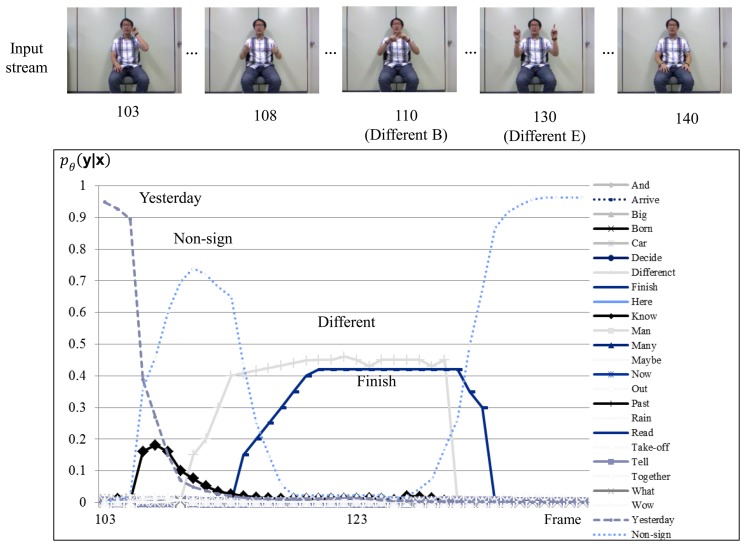
Sign language recognition result for H-CRF**^3^***^D^* using a signed sentence that includes the sign “Different”.

Figure 8 shows the sign recognition results with H-CRF**^3^*^D^***. Hand shape recognition is executed over several frames when the probability of the recognized sign is lower than the threshold, as discussed in Section 3. As shown in the time evolutions of probabilities, the probabilities of the sign “Different” and “Finish” are similar to each other in frames 117 and 129. The probabilities, *P* (*y_i_^t^*), of the signs “Different” and “Finish” are over the threshold in frame 132.

**Figure 9.**
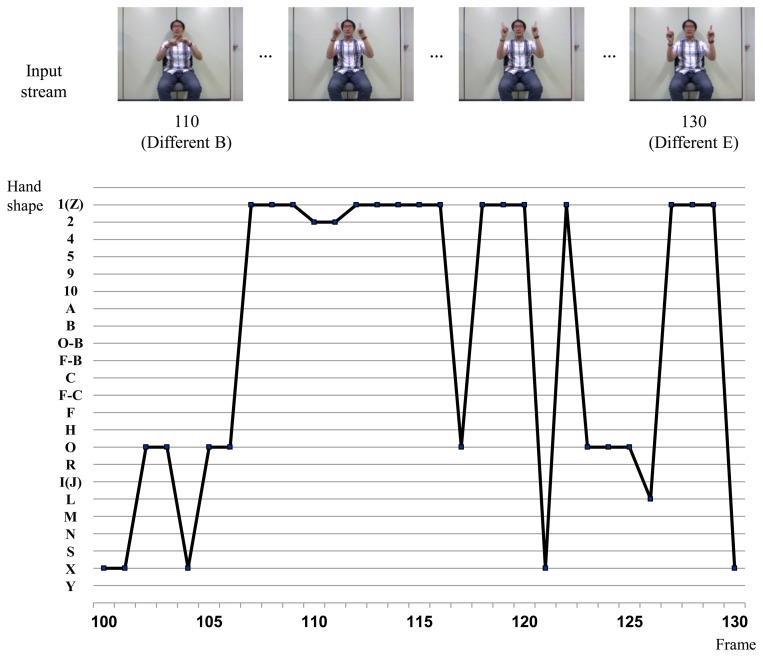
Hand shape recognition results with the signed sentence of Figure 8.

Figure 9 shows the hand shape verification results with the BoostMap embeddings in the sign segment of Figure 8. The frame-wise fingerspelling inference results are presented. The hand appearances of all signs over the threshold are verified as described in Equation (6). Then the sign that has the maximum *V_s_*() is selected, using:

(9) $$y_{i}=\underset{k\in C}{\mathrm{argmax}}\left(V_{S}\left({y_{k}}^{t}\right)\right)$$

where *C* is the set of signs, in which probability *P*(*y_i_^t^*) is over the threshold.

The BoostMap embedding method decreases the insertion and substitution errors by verifying the hand shape; however, it reduces the correct detection rate because of its own classification errors.

## 4. Conclusions and Further Research

Sign language recognition with depth sensors is becoming more widespread. However, it is difficult to detect meaningful signs from a contiguous hand-motion stream because the signs vary in both motion and shape in 3D space. In our work, we recognized meaningful sign language from a contiguous hand-motion stream using a hierarchical CRF framework. The first layer, a T-CRF, is applied to distinguish signs and non-sign patterns. The second layer, a conventional CRF, is applied to distinguish between the shared patterns among the signs.

In this paper, a novel method for recognizing sign language hand gestures was proposed. In order to detect 3D locations of the hands and face, depth information generated with Microsoft's Kinect was used. A hierarchical threshold CRF is also used in order to recognize meaningful sign language gestures using continuous hand motions. Then, the segmented sign was verified with the BoostMap embedding method. Experiments demonstrated that the proposed method recognized signs from signed sentence data at a rate of 90.4%. Near-term future work includes improving the detection accuracy of the upper body components.

## Figures and Tables

**Table 1.** Seven features for recognizing the signer's hand.

**Features**	**Meanings**
*HF_L_*	Position of the left hand with respect to the signer's face
*HF_R_*	Position of the right hand with respect to the signer's face
*HH_L_*	Position of the left hand with respect to the previous left hand
*HH_R_*	Position of the right hand with respect to the previous right hand
*FS_L_*	Position of the left hand with respect to the shoulder center
*FS_R_*	Position of the right hand with respect to the shoulder center
*OH_LR_*	Occlusion of two hands

**Table 2.** Examples of hand shapes for sign language recognition: Categories of hand shapes are described in [1,3].

**Signs**	**Dominant Hand Shape**	**Non-Dominant Hand Shape**
Car (T)	S	S
Past (O)	Open B > Bent B	D.C.
Out (O)	Flat C > Flat O	D.C.

O stands for one-handed sign; T stands for two-handed sign; D.C. means don't care; > Means that the hand shapes of start and end frames of the sign are changed.

**Table 3.** 24 ASL signs used in the vocabulary.

One-handed signs	And, Know, Man, Out, Past, Tell, Yesterday
Two-handed signs	Arrive, Big, Born, Car, Decide, Different, Finish, Here, Many, Maybe, Now, Rain, Read, Take-off, Together, What, Wow

**Table 4.** ASL recognition results.

	**C**	**S**	**I**	**D**	**SER(%)**	**R(%)**
CRF^2D^	185	34	25	21	33.3	77.0
*T*-CRF^2D^ [1]	197	27	24	16	27.9	82.0
H-CRF^2D^ [2]	202	23	15	15	22.0	84.1
H-CRF^3D^	217	12	9	11	13.3	90.4

N is 240; 3D means using features extracted in 3D space; 2D means using features extracted in 2D space.
